# Antiplatelet therapy for patients with COVID-19: Systematic review and meta-analysis of observational studies and randomized controlled trials

**DOI:** 10.3389/fmed.2022.965790

**Published:** 2022-09-07

**Authors:** Xiaolong Zong, Xiao Wang, Yaru Liu, Zhenyu Li, Weiding Wang, Dianjun Wei, Zhuqing Chen

**Affiliations:** ^1^Department of Clinical Laboratory, The Second Hospital of Tianjin Medical University, Tianjin, China; ^2^Institute of Infectious Diseases, The Second Hospital of Tianjin Medical University, Tianjin, China; ^3^Department of Emergency Medicine, The Second Hospital of Tianjin Medical University, Tianjin, China; ^4^Department of Cardiology, The Second Hospital of Tianjin Medical University, Tianjin, China; ^5^Department of Clinical Laboratory, Yanda Hospital, Langfang, China; ^6^Medical Security Center, The No. 983 Hospital of the Joint Service Support Force, Tianjin, China

**Keywords:** coronavirus disease 2019 (COVID-19), thromboembolism, antiplatelet therapy, aspirin, clopidogrel, systematic review, meta-analysis

## Abstract

**Background:**

Hyperinflammation and coagulopathy are hallmarks of COVID-19 and synergistically contribute to illness progression. Antiplatelet agents have been proposed as candidate drugs for COVID-19 treatment on the basis of their antithrombotic and anti-inflammatory properties. A systematic review and meta-analysis that included early observational studies and recent randomized controlled trials (RCTs) was performed to summarize and compare evidence on this issue.

**Methods:**

PubMed, Embase, and the Cochrane Central Register of Controlled Trials (CENTRAL) were searched to identify studies published up to Nov 7, 2021, and the results of registered clinical trials were followed up to Mar 30, 2022. We included RCTs and observational studies assessing the effect of antiplatelet therapy in adult patients with COVID-19. Data on baseline patient characteristics, interventions, controls, and outcomes were extracted by two independent reviewers. The primary outcome was mortality. Data were pooled using a random-effects model.

**Results:**

Twenty-seven studies were included, of which 23 observational studies were pooled in a meta-analysis, and the remaining four RCTs (ACTIV-4B, RECOVERY, ACTIV-4a, and REMAP-CAP) were narratively synthesized. Based on 23 observational studies of 87,824 COVID-19 patients, antiplatelet treatment favors a lower risk of mortality [odds ratio (OR) 0.72, 95% confidence interval (CI) 0.61–0.85; *I*^2^ = 87.0%, *P* < 0.01]. The narrative synthesis of RCTs showed conflicting evidence, which did not support adding antiplatelet therapy to the standard care, regardless of the baseline illness severity and concomitant anticoagulation intensity.

**Conclusion:**

While the rationale for using antiplatelet treatment in COVID-19 patients is compelling and was supported by the combined result of early observational studies, evidence from RCTs did not confirm this approach. Several factors that could explain this inconsistency were highlighted alongside perspectives on future research directions.

## Introduction

Dysregulated inflammation and coagulopathy are hallmarks of severe COVID-19 and contribute to an increased risk of thromboembolic complications and mortality (1–4). Platelets are anucleate cell fragments derived from megakaryocytes that are not simply involved in thrombosis and immune response but also exert a hub function bridging these two processes as a new mechanism, termed immunothrombosis (5–7). The multifaceted role of platelets in immunothrombosis has been well-documented in previous literature and further highlighted in the current COVID-19 situation (8–11). A hyperactive platelet phenotype, as characterized by increased platelet surface markers [e.g., P-selectin, platelet Factor 4 (PF4), and CD40L], platelet-derived soluble mediators [e.g., thromboxane B2 (TxB2) and 5-hydroxytryptamine (5-HT)], and platelet homotypic and heterotypic aggregates, has been extensively identified in COVID-19 patients (9, 12–16). Likewise, data from transcriptome and proteome analyses indicate that platelet hyperactivity is a predominant cellular signature in response to SARS-CoV-2 infection (17, 18), thus suggesting a possible role of platelets in this novel viral disease. Moreover, activated platelets can trigger the formation of neutrophil extracellular traps (NETs) (19, 20), which have recently been recognized as pivotal players in thrombosis (21–23). Autopsy reports of COVID-19 patients revealed microthrombi with platelet and NET deposition in inflamed lung tissues, along with endothelial disruption (23–25).

Given the possible role of hyperactive platelets in the pathological mechanism of COVID-19, antiplatelet agents, such as aspirin and P2Y12 inhibitors, have been proposed as a potential treatment strategy for COVID-19 patients on the basis of their antithrombotic and anti-inflammatory properties (26–28). Additionally, significant thrombotic events have been observed despite anticoagulant treatment in clinical trials, implying that antiplatelet agents could be potential candidates for additional adjunctive antithrombotic therapy (29–31). In fact, an association between antiplatelet drug use and improved outcomes for COVID-19 patients has been reported in early observational studies (32–34). However, recently completed RCTs failed to confirm the effectiveness of antiplatelet treatment in preventing COVID-19 progression. While RCTs are considered to be more reliable than observational studies in evaluating interventions, the latter has helped us establish an initial foundation, which is particularly significant in an urgent situation (35). In the present circumstances, there is a need for findings to be assessed in the context of existing evidence in order to ensure reasonable interpretation of all studies (36). Here, we perform a systematic review and meta-analysis that included both RCTs and observational studies to provide an overview of existing evidence on antiplatelet therapy in patients with COVID-19. Furthermore, several study elements (e.g., baseline illness severity, the timing of antiplatelet therapy, and concomitant anticoagulation intensity) that might contribute to discrepancies among current lines of evidence and should be taken into consideration in future research are discussed.

## Methods

This systematic review was performed following the PRISMA statement (37). The study protocol is provided in Supplementary material 1. Briefly, PubMed, Embase, and Cochrane CENTRAL were searched to identify studies published up to Nov 7, 2021, and the results of registered clinical trials were followed up to Mar 30, 2022. Details of the search strategies are provided in Supplementary material 1. The inclusion criteria were adult COVID-19 patients confirmed by laboratory testing, administration of antiplatelet therapy at any time or dose, comparison between patients with and without antiplatelet therapy, and availability of English or Chinese full texts. Studies involving patients with a particular illness or emergency conditions were excluded (e.g., cancer and pregnancy). When studies had significant overlapping data, the most comprehensive study was included.

The results of observational studies and RCTs were separately synthesized and compared (35). For pooled analysis, we selected all-cause mortality as the primary outcome for effect estimates. Considering that all the included studies for quantitative pooled analysis were retrospective in design, the odds ratio (OR) was used as the common measure of association across studies. Hazard ratios (HRs) and relative risks (RRs) were directly considered as ORs. A random-effect model was selected to account for clinical heterogeneity. Heterogeneity across studies was assessed using the Q statistic with its *P*-value and *I*^2^ statistic (38). Subgroup analyses were conducted to investigate variation in estimates according to original effect size, study center, illness severity, antiplatelet drugs, the timing of drug administration, and concomitant anticoagulant use. Sensitivity analysis was performed on the primary outcome by omitting one study at a time to assess the robustness of the results (39). A funnel plot was drawn to assess publication bias. The quality of the included observational studies for meta-analysis was evaluated following the Newcastle–Ottawa scale (NOS) by two independent reviewers (40). Studies with NOS scores of 8 or 9, 6 or 7, and < 6 were judged as having a low, medium, and high risk of bias, respectively. Discrepancies in data extraction and quality assessment were resolved through discussion with a third author. Statistical analyses were performed using RStudio software.

## Results

### Study identification

**Figure 1:**
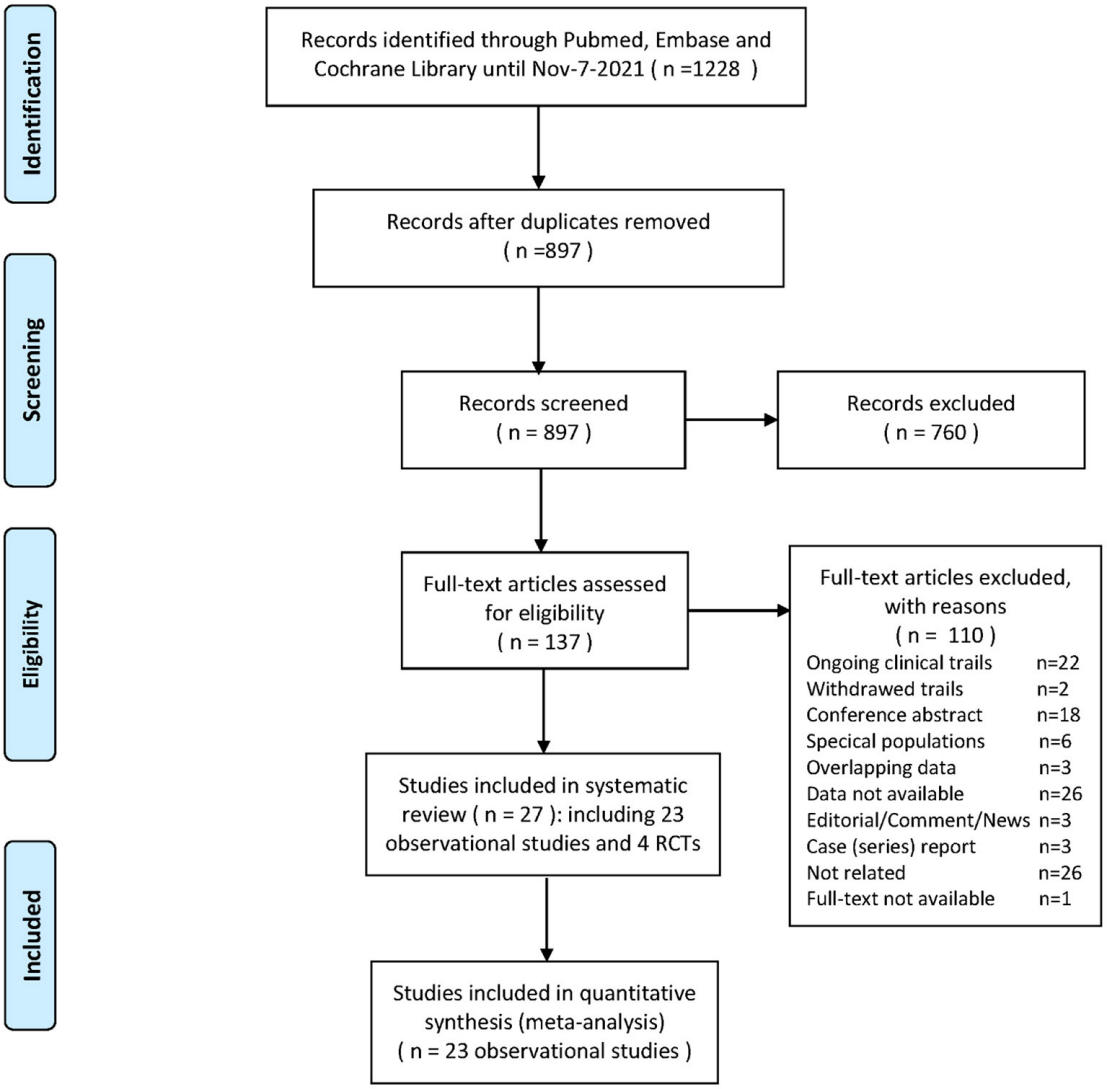
Flowchart of the systematic review.

Our search yielded 1,228 records. After initial screening and full-text review, 23 observational studies (41–63) and 4 RCTs (64–67) (ACTIV-4B, RECOVERY, ACTIV-4a, and REMAP-CAP) were finally included for evidence synthesis and comparison (Figure 1). Observational studies were mostly performed in the first half of 2020, and RCTs were subsequently conducted between late 2020 and early 2021. For observational studies, the overall risk of bias was determined to be medium (Supplementary Table 1). Adjusted estimates could be determined for all observational studies even though the adjusted factors were slightly different. Among all studies, aspirin is the most common antiplatelet drug. Tables 1, 2 show details of the included observational studies and RCTs, respectively.

**Table 1:** Details of observational studies included in this meta-analysis of the association between antiplatelet treatment and mortality.

**References**	**Time of patient inclusion**	**Region**	**Center**	**Population**	***N***	**Age (year)**	**Male sex**	**Drugs**	**Endpoint**	**Events rate**	**Adjusted factors**	**NOS**
Aydinyilmaz et al. (41)	Mar to Dec 2020	Turkey	S	Severely ill Inpatients	373	E: 73.9 ± 0.9 C: 69.1 ± 1.9	E: 72.9% C: 58.0%	Aspirin	In-hospital mortality	–	Male gender, diabetes, hypertension	7
Chow et al. (42)	Feb to Apr 2020	United States	M	Inpatients	17,347	E: 72 (64–80) C: 72 (64–80)	E: 54.5% C: 53.3%	Multiple^*^	In-hospital mortality	20.5%	Age, male, race, BMI, comorbidities, medications	8
Corrochano et al. (43)	Mar to May 2020	Spain	S	Inpatients	1,443	66.5 ± 17.1	53.2%	Multiple	28 d mortality	19.3%	Sex, age, comorbidities	8
Fröhlich et al. (44)	Feb to Apr 2020	Germany	M	Inpatients	5,971	E:79 (69–84) C: 65 (52–79)	E: 63.8% C: 51.1%	Multiple	All-cause mortality or ventilation	27.5%	Age, gender, and comorbidities	8
Gupta et al. (45)	Feb to May 2020	United States	S	Inpatients	2,626	–	–	P2Y12 inhibitor	30 d mortality	–	Age, sex, BMI, comorbidity, medications	7
Haji Aghajani et al. (46)	Mar 2019 to Jul 2020	Iran	S	Severely ill inpatients	991	61.6 ± 17.0	54.9%	Aspirin	In-hospital mortality	25.8%	Age, sex, BMI, comorbidity, medications	7
Ho et al. (47)	Feb to Jul 2020	United States	M	Outpatients	27,824	E: 66 (55–77) C: 41 (30–53)	E: 53.0% C: 48.0%	Multiple	Mortality	3.3%	Age, sex, race, BMI, comorbidities	8
Izzi-Engbeaya et al. (48)	Mar to Apr 2020	UK	M	Inpatients	889	65.8 ± 17.5	60.1%	–	Death and/or ICU admission	36.0%	Age, sex, race, comorbidity, Laboratory and clinical parameters, and medications	6
Liu et al. (49)	Jan to Mar 2020	China	S	Inpatients	48	E: 69 (61–76) C: 74 (65–79.5)	E: 58.3% C: 70.8%	Aspirin	30 d mortality	16.7%	Age, sex, comorbidities, Laboratory and clinical parameters, and medications	8
Matli et al. (50)	Apr 2020 to Jan 2021	Lebanon	S	Inpatients	146	E: 66.2 ± 13.8 C: 59.6 ± 17.0	E: 67.4% C: 58.8%	Multiple	In-hospital mortality	14.1%	Age, sex, smoking, weight, comorbidity, medications	8
Meizlish et al. (51)	Mar to Jun 2020	United States	M	Inpatients	638	–	63.3%	Aspirin	In-hospital mortality	–	Age, sex, max D-dimer, comorbidities, medications	8
Merzon et al. (52)	Feb to Jun 2020	Israel	M	Inpatients	112	–	–	Aspirin	In-hospital mortality	6.3%	Age, sex, smoking, comorbidity, medications	7
Mura et al. (53)	–	30 countries	M	Severely ill inpatients	527	–	–	Aspirin	Mortality	31.3%	Age, gender	6
Osborne et al. (54)	Mar to Aug 2020	United States	M	Inpatients	12,600	E: 67.4 ± 10.7 C: 67.2 ± 11.1	E: 95.2% C: 96.6%	Aspirin	30 d mortality	7.4%	Age, gender, and Care Assessment Needs (CAN) score	8
Pan et al. (55)	Mar to Apr 2020	United States	S	Inpatients	762	E: 69.6 ± 12.5 C: 58.5 ± 16.2	E: 60.3% C: 54.3%	Multiple	28 d mortality	~20%	Age, sex, BMI, smoking, comorbidities	8
Russo et al. (56)	Feb to Apr 2020	Italy	M	Inpatients	192	67.7 ± 15.2	59.9%	Multiple	In-hospital mortality	18.5%	Age, smoke, comorbidities	8
Sahai et al. (57)	Mar to May 2020	United States	M	Outpatients	496	E: 68.5 ± 13.6 C: 69.5 ± 14.1	E: 56.5% C: 59.5%	Aspirin	In-hospital mortality	14.3%	Age, sex, race, smoking, plateletgs, and comorbidities	7
Santoro et al. (58)	Jan to May 2020	7 countries	M	Inpatients	7,716	64 ± 17	58.0%	Multiple	In-hospital mortality	18.0%	Age, sex, comorbidities, invasive ventilation, medications	8
Sisinni et al. (59)	Feb to Apr 2020	Italy	M	Inpatients	984	72 [62–81]	69.0%	Multiple	30 d mortality or respiratory support upgrade	72.0%	Age, male gender, hypertension, glucocorticoid therapy	8
Soldevila et al. (60)	Mar to Jun 2020	Spain	M	Inpatients	1,306	86.7 ± 7.3	28.7%	Multiple	30 d mortality	24.4%	Age, gender, comorbidities, Barthel score, frailty score, medications	8
Terlecki et al. (61)	Mar to Oct 2020	Poland	S	Inpatients	1,729	63 [50–75]	51.2%	Multiple	In-hospital mortality	12.9%	Age, gender, comorbidities, medications	8
Tremblay et al. (62)	Mar to Apr 2020	United States	M	Inpatients	1,064	E: 61.2 ± 10.9 C: 63.0 ± 12.2	54.9% 57.5%	–	In-hospital mortality	15.0%	Age, sex, race, Charlson comorbidity index and obesity	8
Zhao et al. (63)	Feb 2020 to Mar 2021	United States	M	Severely ill inpatients	2,070	65 ± 16	58.8%	Aspirin	In-hospital mortality	29.0%	Age, sex, smoking, BMI, comorbidity, laboratory indices, vital signs, medications	8

* Two or more antiplatelet drugs were together defined as exposure, with aspirin plus P2Y12 inhibitors being most common among studies. The ages of the study populations were expressed as the mean ± standard deviation or median [interquartile range].

BMI, body mass index; E, exposure group; C, control group; –, Data not reported or not calculable; NOS, Newcastle–Ottawa Scale.

**Table 2:** Details of RCTs investigating the effect of antiplatelet treatment for patients with COVID-19.

**Study**	**Study time frame**	**Number of centers/country**	**Population**	***N***	**Median/mean age (year)**	**Male (%)**	**Antiplatelet treatment**	**Primary outcome**
ACTIV-4B (64)	Sep 2020– Jun 2021	52/USA	Symptomatic but clinically stable outpatients	328	54.0	41.8	Aspirin (81 mg)	A composite of all-cause mortality, thromboembolic events or hospitalization for cardiovascular or pulmonary cause
RECOVERY (65)	Nov 2020– Mar 2021	171/UK, Indonesia, Nepal	Adult hospitalized patients	14 892	59.2	61.8	Aspirin (150 mg)	28 d mortality
								
ACTIV-4a (66)	Feb–Jun 2021	60/Brazil, Italy, Spain, USA	Non-critically ill hospitalized patients	562	52.7	58.5	P2Y12 inhibitors	Organ support-free days
REMAP-CAP (67)	Oct 2020– Jun 2021	105/Canada, France, Germany, India, Italy, Nepal, the Netherlands, UK	Critically ill hospitalized patients	1 549	57.0	66.4	Aspirin (75–100 mg) or P2Y12 inhibitors	Respiratory and cardiovascular organ support–free days to day 21

### Meta-analysis of observational studies

**Figure 2:**
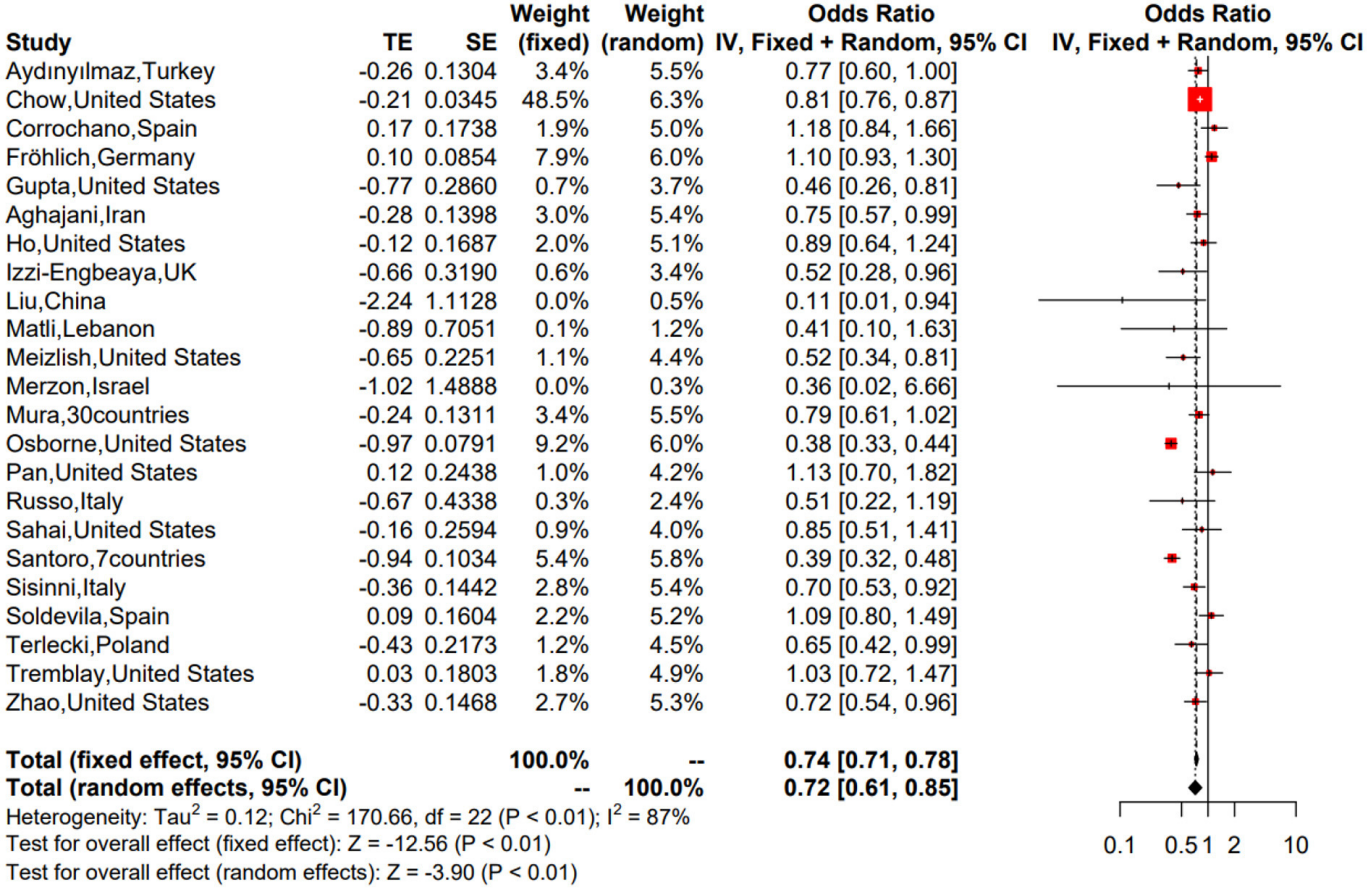
Forest plot of the 23 included observational studies.

**Figure 3:**
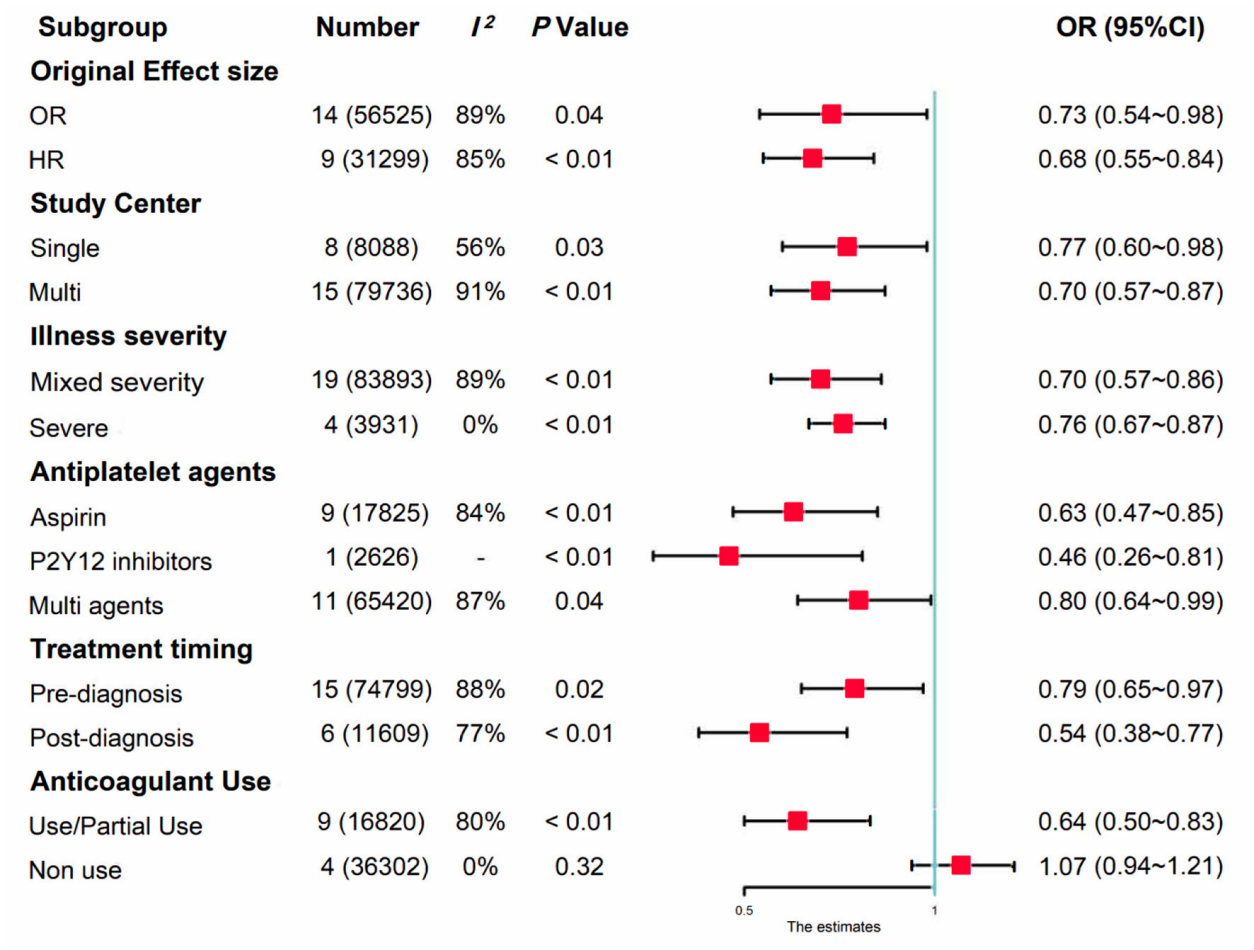
Summary of the subgroup analyses in observational studies.

We first performed a meta-analysis of the 23 included observational studies and obtained a combined OR of 0.72 (95% CI: 0.61–0.85), suggesting that antiplatelet treatment favors a lower risk of mortality in patients with COVID-19 (Figure 2). Although significant heterogeneity was evident (*P* < 0.01, *I*^2^ = 87%), the combined estimates were consistent in the random- and fixed-effect models, and the sensitivity analysis suggested that our result was stable (Supplementary Figure 1), probably driven by a large number of participants (*n* = 87,824). To investigate the variation of combined evidence among observational studies and improve comparability with RCTs, pre-specified subgroup analyses were further conducted. All results of the subgroup analyses are summarized in Figure 3 (forest plots are shown in Supplementary Figures 2–7). The only significant antiplatelet treatment-covariate interaction identified in subgroup analyses was concomitant anticoagulant use, with OR = 0.64 (95% CI: 0.50–0.83) among patients with anticoagulant use (including partial use) and OR = 1.07 (95% CI: 0.94–1.21) among patients without anticoagulation treatment. There was no evidence to suggest a differential treatment effect for any other subgroups. The asymmetric shape of the funnel plot shows some evidence of publication bias among the evaluated studies (Supplementary Figure 8).

### RCTs

Currently, one outpatient trial (ACTIV-4B) (64) and three inpatient trials (RECOVERY, ACTIV-4a, and REMAP-CAP) (65–67) have released their results. As there was obvious heterogeneity in the study population, antiplatelet treatment regimen, and concomitant anticoagulation intensity among these studies, their results were narratively synthesized (Table 2). The ACTIV-4B trial aimed to assess whether antiplatelet therapy (aspirin 81 mg) can safely reduce major adverse cardiopulmonary outcomes among symptomatic but clinically stable outpatients with COVID-19. The study was terminated early because of an event rate (0.7%) lower than anticipated and no evidence of efficacy when comparing aspirin with placebo.

RECOVERY (65) is the current largest randomized study investigating the effect of antiplatelet therapy in COVID-19, with 14,892 participants from 171 centers. This study found that in a mixed population of patients with mild, moderate, and severe COVID-19, adding 150 mg aspirin to standard care did not reduce 28-day mortality [relative risk (RR) = 0.96, 95% CI: 0.89–1.04] or the probability of progression to the composite of invasive mechanical ventilation or death (RR = 0.96; 95% CI: 0.90–1.03).

In recently completed multiplatform trials (ATTACC, ACTIV-4a, and REMAP-CAP), therapeutic-dose heparin vs. conventional thromboprophylaxis has been found to improve organ support-free days in hospitalized non-critically ill patients (30) but is not beneficial for critically ill patients (29). Subsequently, the ACTIV-4a trial (66) tested whether the addition of a P2Y12 inhibitor to anticoagulant therapy would further change clinical outcomes in non-critically ill patients hospitalized for COVID-19. After 562 patients completed the trial, no significant differences were found in the primary outcome (the composite of organ support-free days evaluated on an ordinal scale combined with in-hospital death) or in the secondary outcome (the composite of major thrombotic events or death by 28 days).

In parallel with ACTIV-4a, the REMAP-CAP trial (67) aimed to examine the add-on effect of antiplatelet therapy [aspirin, 75–100 mg; *n* = 565 or P2Y12 inhibitors (clopidogrel, 75 mg; ticagrelor, 60 mg; or prasugrel, 60 mg); *n* = 455] alongside prophylactic dose anticoagulation in severe COVID-19 patients. First, this trial observed equivalence between the aspirin and P2Y12 inhibitor groups (OR = 1.00; 95% CI, 0.8–1.27; >90% posterior probability of equivalence). In a subsequent adaptive pooled analysis of the two antiplatelet treatment groups in comparison with controls, the median for organ support-free days was 7 in both the antiplatelet and control groups (median-adjusted OR = 1.02; 95% CI, 0.86–1.23; 95.7% posterior probability of futility). Although a modest benefit on the secondary endpoint of 90-day mortality was determined (HR = 1.22; 95% CI, 1.06–1.40; 99.7% posterior probability of efficacy), the median number of organ support-free days was again equal (14 days) among survivors in both groups. Additionally, the authors reported a small but certain increased risk of major bleeding in the antiplatelet group (2.1 vs. 0.4%; adjusted OR = 2.97; 95% CI, 1.23–8.28; 99.4% probability of harm).

## Discussion

This systematic review summarized and compared current evidence regarding antiplatelet treatment for patients with COVID-19. The combined effect estimates of observational studies suggested that antiplatelet therapy favors a lower risk of mortality, and the results were consistent in all pre-specified subgroup analyses in addition to those based on anticoagulant use. Nevertheless, subsequent RCTs did not confirm this association. A series of well-conducted randomized studies found no additional effect when adding antiplatelet therapy to standard care, regardless of baseline illness severity and concomitant anticoagulation intensity. The reason for this inconsistency may be multiple. First, since all observational studies included for pooled analysis were retrospective in design, selection bias might have occurred in the selection of exposed subjects according to an antecedent prescription of antiplatelet medication. Additionally, while adjusted estimates could be determined for all studies, potential cofounding associated with both exposure and outcome cannot be definitively excluded (68, 69).

In addition to the limitations ascribed to the study design *per se*, another noteworthy factor is the timing of antiplatelet treatment. For most observational studies (41–45, 47, 52, 54–57, 59–62) included for evidence synthesis, antiplatelet therapy was initiated before COVID-19 diagnosis in contrast to during hospitalization in RCTs (65–67). Possibly, the baseline suboptimal platelet reactivity due to prior chronic antiplatelet therapy restrains illness progression and aggravation. At the time of hospitalization because of moderate or severe illness, platelet activation may have already reached a maximum level, for which antiplatelet treatment is too late (66). Additionally, as mentioned above, the rationale for antiplatelet medication in COVID-19 is based on the antithrombotic and anti-inflammatory properties. However, there is evidence that the distribution of platelets is not limited only to intravascular compartments but also to alveolar translocation (70–73). Moreover, platelets differentially bind to neutrophils and Treg cells at distinct time points to orchestrate both the initiation and resolution of pulmonary inflammation. These interactions prevent excessive lung damage after infection (70). In sum, platelets still offer a candidate treatment target for infection-related thrombosis, yet the treatment timing may be of great relevance and warrant further investigation at the clinical level.

Among currently completed RCTs, ACTIV-4B is the only outpatient trial. In addition to its negative finding, this trial also reported a markedly low rate of events (a composite of all-cause mortality, thromboembolism, myocardial infarction, stroke, or hospitalization for cardiovascular or pulmonary cause), namely, 0.7% among the study populations, which is much lower than that reported by early epidemiological data (64). The significant decline in adverse event incidence among mildly ill outpatient populations could partially be attributed to aggressive vaccination and progress in medical care since the pandemic outbreak (74, 75). Correspondingly, the recently updated COVID-19 treatment guidelines recommend against the use of anticoagulants and antiplatelet therapy in the outpatient setting, unless the patient has other indications for the therapy (76). Another ongoing trial (OLA COVID; NCT04937088) (77) that tests whether a novel, liquid aspirin formulation can reduce COVID-19-related hospitalizations in old populations will provide more evidence on this issue.

For hospitalized patients with COVID-19, thrombotic complications have been reported to be common despite conventional thromboprophylaxis and therapeutic anticoagulation (31). In this setting, the RECOVERY, ACTIV-4a, and REMAP-CAP trials sought to examine the additional effect and risk of antiplatelet treatment on the basis of thromboprophylaxis and anticoagulation therapy. Overall, the results of these well-conducted trials found no additional effect when antiplatelet therapy was added to anticoagulation (mostly Low-molecular-weight heparin, LMWH) in hospitalized patients with COVID-19, despite a slightly increased risk of bleeding. The reason for these negative results is not obvious. A possible explanation is that the anticipated antithrombotic effect of antiplatelet drugs was partially masked by LMWH (78).

While the mechanism of COVID-19-related coagulopathy has not yet been elucidated, a major cause is tissue factor overexpression on the surface of damaged endothelial cells and immune cells, which further initiates coagulation cascades and leads to thrombin generation (8). This opinion can be supported by anticoagulation trials that found the superiority of heparin/LMWH by targeting thrombin. However, thrombin is not only a central enzyme involved in coagulation cascades but also a potent inducer of platelet activation (78, 79). In a more recent study, the TF/thrombin pathway was found to be pivotal for platelet activation in an *ex vivo* SARS-CoV-2 infection model (80). The authors concluded that TF activity from SARS-CoV-2-infected cells activates thrombin, which signals to protease-activated receptors (PARs) on platelets (80). Taken together, it is plausible to speculate that the key upstream pathway that promotes platelet activation during SARS-CoV-2 infection is inhibited by heparin through disturbing thrombin ligation to platelet Glycoprotein Ib (GP Ib) and PARs (78, 80), whereby the anticipated antithrombotic effects of aspirin and P2Y12 inhibitors in the above trials were diluted.

To date, our successful experience in the combined use of heparin and antiplatelet agents is almost confined to thrombotic disease, with most valid evidence in arterial thrombosis, yet, under the premise that antiplatelet treatment *per se* is effective (81). Whether antiplatelet therapy alone can prevent illness progression for hospitalized patients with COVID-19 is still unclear. This question is difficult to clarify in future trials, as it is unethical to abrogate proven beneficial anticoagulation for patients to measure the effect of single antiplatelet therapy. Alternatively, early observational studies could shine a light on this issue. In the subgroup analysis of observational studies by anticoagulant intensity, we identified four studies (43, 44, 47, 62) including 36,302 patients without anticoagulant use. The combined OR of 1.07 (95% CI, 0.94–1.21; *I*^2^ = 0%, *P* = 0.32) suggested that single antiplatelet treatment is not associated with lower mortality (see Figure 3). However, this result may be limited by the lack of sufficient direct comparisons and should be regarded with extreme caution. In contrast to targeting platelets *per se*, there is an ongoing arm of the ACTIV-4a trial that aims to test whether inhibiting the cross-talk between platelets and immune as well as endothelial cells, by using Crizanlizumab (82, 83) or sodium-glucose cotransporter-2 (SGLT2) inhibitor (84), will further improve the hypercoagulable state of patients with COVID-19, and the results are eagerly anticipated.

## Conclusion

This paper provides an overview of existing evidence on antiplatelet therapy for patients with COVID-19. In summary, while the rationale for using antiplatelet drugs to prevent COVID-19 progression is compelling and was supported by combined evidence from early observational studies, recently completed RCTs do not support this approach. The consistent negative results of such RCTs have supplied more valid evidence against adding antiplatelet therapy to standard care for COVID-19 patients in either community or hospital settings. In terms of directions for future study, the optimal antithrombotic regimen for patients with COVID-19 should be individualized (85) and guided by biomarkers, such as urinary 11-dehydrothromboxane B2, platelet reactivity, platelet-platelet aggregates, and platelet-leukocyte aggregates detected by new microscopic techniques (16, 31). Moreover, several factors that could explain the inconsistency among the current evidence and advocate for further investigation were highlighted in the current review.

## Data availability statement

The datasets used during the current study are available from the corresponding author upon reasonable request.

## Author contributions

XZ contributed to the conception and design of the study, acquisition of data, analysis and interpretation of the data, and drafting of the manuscript. ZL, WW, DW, and ZC reviewed and revised the manuscript. XW, YL, and ZL contributed to the acquisition as well as to the analysis and interpretation of the data. All authors gave final approval and agree to be accountable for all aspects of the work ensuring integrity and accuracy.

## Funding

This work was supported by the Tianjin Health Science and Technology Project (KJ20092), the Youth Training Program, the Second Hospital of Tianjin Medical University (2019ydey28), Tianjin Health and Family Planning Industry Young Medical Talents Project, Integrated Chinese and Western Medicine Project (2021207), and Hebei Health Science and Technology Projects (20191066 and 20191061).

## Conflict of interest

The authors declare that the research was conducted in the absence of any commercial or financial relationships that could be construed as a potential conflictof interest.

## Publisher's note

All claims expressed in this article are solely those of the authors and do not necessarily represent those of their affiliated organizations, or those of the publisher, the editors and the reviewers. Any product that may be evaluated in this article, or claim that may be made by its manufacturer, is not guaranteed or endorsed by the publisher.

## Abbreviations

5-HT: 5-Hydroxytryptamine
CI: Confidence intervals
COVID-19: Coronavirus Disease
GP Ib: Glycoprotein Ib
HR: Hazard ratio
LMWH: Low-molecular-weight heparin
NETs: Neutrophil extracellular traps
NOS: Newcastle–Ottawa Scale
OR: Odds ratio
PARs: Protease-activated receptors
PF4: Platelet Factor 4
RCTs: Randomized controlled trials
RR: Relative risk
SARS-CoV-2: Severe acute respiratory syndrome coronavirus 2
SGLT2: Sodium-glucose cotransporter-2
TxB2: Thromboxane B2.
