# Supplementary material for: Antiplatelet therapy for patients with COVID-19: Systematic review and meta-analysis of observational studies and randomized controlled trials
Source: Front Med (Lausanne). 2022 Sep 7;9:965790. doi: 10.3389/fmed.2022.965790 (PMC9490267; doi:10.3389/fmed.2022.965790)
Supplement: Supplementary file 1 [file Data_Sheet_1.pdf]

Supplementary file 1 for

Antiplatelet Therapy for Patients with COVID-19: Systematic Review and Meta-Analysis of Observational Studies and Randomized Controlled Trials

STUDY PROTOCOL

Background

Hyperinflammation and coagulation disorders are hallmarks of severe COVID-19 and increase the risk of thromboembolic complications, a major cause of morbidity and mortality in COVID-19 patients [1, 2]. Platelets play a pivotal role in thrombosis and inflammation, they also actively link the two processes, creating a new mechanism called thrombo-inflammation, or immune-thrombosis [3, 4]. During SARS-COV-2 infection, platelets became hyperactive as shown by the increased surface P-selectin expression and increased formation of circulating platelet–neutrophil aggregates (PNAs) via binding with PSGL-1 [5-9]. The generation of PNAs recruit neutrophils to damaged lung capillaries [5-7]. Thrombotic events in COVID-19 may be attributed to platelets augmenting inflammation through the generation of NETs and pro-coagulant platelets, increased aggregates (e.g., PNAs) and the release of bioactive substances [10-13].

Given the close association between hyperactive platelets and COVID-19, antiplatelet agents, such as aspirin and P2Y12 receptor antagonist, have been proposed as potential treatment strategy for COVID-19 patients on the basis of their antithrombotic and anti-inflammation properties [14, 15]. In fact, this hypothesis has been verified in many well-designed observational studies [16-21]. However, recently completed RCTs (RECOVERY, ACTIV-4a) indicated no significant beneficial effect of antiplatelet drugs in both mild and critical COVID-19 populations [22, 23]. Considering there is still a lack of consensus on the use of antiplatelet agents for COVID-19 treatment at present, and many clinical trials are still ongoing. We make a systematic review on the current evidence from RCTs and observational studies that investigating the effect of antiplatelet treatment in COVID-19 patients.

Objectives

To investigate the association of antiplatelet treatment with outcomes of patients with COVID-19.

PICOS

Population: Patients with COVID-19
Exposures: Antiplatelet drugs
Comparison: Without antiplatelet drugs use
Outcome:
Primary outcome: All-cause mortality
Secondary outcome: Thromboembolic events or Organ support-free days
Study design: Observational studies (Cohort/Case-control studies) and Random control trials (RCTs)

Search Strategy

Search databases: PubMed (Medline), Embase, and Cochrane CENTRAL

PubMed (Medline) （**Until 2021-11-07**）

#1 "COVID 19" [Mesh]

#2 "Corona Virus Disease 2019"

#3 "coronavirus disease-19"

#4 "SARS Coronavirus 2 Infection"

#5 "2019 novel coronavirus Disease"

#6 "2019-nCoV Disease"

#7 #1 OR #2 OR #3 OR #4 OR #5 OR #6

#8 "Platelet Aggregation Inhibitors" [Mesh]

#9 "antiplatelet"

#10 "antiplatelet drugs"

#11 "Platelet Antiaggregants"

#12 "Platelet Aggregation Inhibitor"

#13 "aspirin"

#14 "acetylsalicylic acid"

#15 "clopidogrel"

#16 "prasugrel"

#17 "ticagrelor"

#18 "ticlopidine"

#19 "cilostazol"

#20 "dipyridamole"

#21 "tirofiban"

#22 "eptifibatide"

#23 "abciximab"

#24 "anagrelide"

#25 "vorapaxar"

#26 "atopaxar"

#27 #8 OR #9 OR #10 #11 OR #12 OR #13 OR #14 OR #15 OR #16 OR #17 OR #18 OR #19 OR #20 OR #21 OR #22 OR #23 OR #24 OR #25 OR #26

#28 #7 AND #27

Embase (Until 2021-11-07)

#1 'coronavirus disease 2019':ab,ti

#2 'covid 19':ab,ti

#3 'sars coronavirus 2 infection':ab,ti

#4 'sars-cov-2 infection':ab,ti

#5 '2019 novel coronavirus disease':ab,ti

#6 '2019-ncov disease':ab,ti

#7 #1 OR #2 OR #3 OR #4 OR #5 OR #6

#8 'antithrombocytic agent':ab,ti

#9 'platelet aggregation inhibitor':ab,ti

#10 'antiplatelet':ab,ti

#11 'antiplatelet drugs':ab,ti

#12 'platelet antiaggregants':ab,ti

#13 'aspirin':ab,ti

#14 'acetylsalicylic acid':ab,ti

#15 clopidogrel:ab,ti

#16 prasugrel:ab,ti

#17 ticagrelor:ab,ti

#18 ticlopidine:ab,ti

#19 cilostazol:ab,ti

#20 dipyridamole:ab,ti

#21 tirofiban:ab,ti

#22 eptifibatide:ab,ti

#23 abciximab:ab,ti

#24 anagrelide:ab,ti

#25 vorapaxar:ab,ti

#26 atopaxar:ab,ti

#27 #8 OR #9 OR #10 #11 OR #12 OR #13 OR #14 OR #15 OR #16 OR #17 OR #18 OR #19 OR #20 OR #21 OR #22 OR #23 OR #24 OR #25 OR #26

#28 #7 AND #27

Cochrane CENTRAL (Until 2021-11-06)

#1 MeSH descriptor: [COVID-19] this term only

#2 COVID 19

#3 Coronavirus Disease 19

#4 Coronavirus Disease 2019

#5 2019 nCoV Disease

#6 2019 nCoV Infection

#7 SARS CoV 2 Infection

#8 #1 OR #2 OR #3 OR #4 OR #5 OR #6 OR #7

#9 MeSH descriptor: [Platelet Aggregation Inhibitors] this term only

#10 Platelet Inhibitors

#11 Platelet Antagonists

#12 Antiplatelet

#13 aspirin

#14 acetylsalicylic acid

#15 clopidogrel

#16 prasugrel

#17 ticagrelor

#18 ticlopidine

#19 cilostazol

#20 dipyridamole

#21 tirofiban

#22 eptifibatide

#23 abciximab

#24 anagrelide

#25 vorapaxar

#26 atopaxar

#27 #9 OR #10 OR #11 OR #12 OR #13 OR #14 OR #15 OR #16 OR #17 OR #18 OR #19 OR #20 OR #21 OR #22 OR #23 OR #24 OR #25 OR #26

#28 #8 AND #27 in Trials

Inclusion Criteria

1) Inclusion of adult patients with COVID-19
2) Administration of antiplatelet therapy at any time or dose
3) Comparison between patients with and without antiplatelet therapy
4) Observational cohort studies, cross-sectional studies
5) English or Chinese version full-text provided

Exclusion Criteria

1) Included patients without Laboratory confirmed
2) Studies focus on specific populations, eg. Pregnant women, children, or patients

comorbid with a particular disease (e.g., cancer, cardiovascular disease, etc.)

3) Endpoint events not clearly defined

4) Overlapping data across studies

5) Case (series) report

Selection of Studies

Two authors will independently screen titles and abstracts of searched records to select potentially eligible studies for full-text review. Any study identified as potentially eligible by either reviewer will be kept for full-text review. Authors will manually review the references of selected review articles to find other relevant studies. Two authors will separately review the full text of potentially relevant studies to identify eligible studies. All disagreements will be resolved by discussion with a third author.

Data Extraction

Data collection forms will be designed by the study team in Microsoft Excel. All data will be extracted and recorded by two authors independently. Arguments will be settled by discussion. Authors of studies will be contacted for further information as needed.

The following variables will be recorded:

Study characteristics: First author, time for objects enrollment, study type, number of study center, location of study centers, study population, sample size, antiplatelet drug, anticoagulation medications, study outcome(s), adjusted confounders

Exposure: Using antiplatelet drug(s) at any time or dose

Baseline characteristics**:** age of population, male/female ratio, baseline illness severity

Outcome: Number of events or Odds ratio (OR) or Hazard ratio (HR) value of antiplatelet agent(s) exposure to predict adverse endpoint events

Table 1 Data extraction form

**Studies**			
Author			
City/Country			

Time for enrollment			
Population			
N			
Male			
Age			
Drugs			
**Primary Endpoint**			
Events rate			
Follow-up time			
Newcastle-Ottawa Scale			
Effect size			
Value (95%CI)			
Adjusted factors			
**2nd Outcome1**			
Effect size			
Value (95%CI)			
Adjusted factors			
**2nd Outcome2**			
Effect size			
Value (95%CI)			
Adjusted factors			

Quality Assessment

Risk of bias

Randomized studies: Study methodological quality will be assessed with the Cochrane Collaboration Risk of Bias Tool [24]. Each study will be evaluated as having a low risk, high risk, or unclear risk of bias in the following areas: (1) sequence generation, (2) allocation concealment, (3) blinding of participants/personnel/outcome assessors, (4) incomplete outcome data, (5) selective outcome reporting, and (6) other sources of bias.

Observational studies: Quality will be assessed using the Newcastle-Ottawa Scale (NOS) [25]. Nine stars NOS for observational cohort studies will be applied. Each study will be evaluated on a 9-point scale in the domains of patient selection, comparability,

exposure and outcome. We judge observational studies with NOS score of nine or eight stars as low risk bias, seven or six stars as median risk of bias, and below six stars as high risk of bias.

Quality of evidence

Grading of Recommendations Assessment, Development, and Evaluation (GRADE) will be used to make judgments about quality of evidence for each outcome [26]. GRADE presents a systematic and transparent framework for clarifying questions, determining the outcomes of interest, summarizing the evidence that addresses a question, and moving from the evidence to a recommendation or decision [26]. We will assess the quality of evidence as high, moderate, low, or very low using GRADE profiler 3.6 (GRADEpro; McMaster University 2014, Hamilton, Canada)

Data Analysis

Randomized and observational studies will be analyzed separately.

Randomized studies**:** A odds ratio (OR) with 95% confidence interval (CI) will be calculated for each study and combined using the number of patients with and without the outcome in exposed and non-exposed groups.

Observational studies**:** An adjusted OR with 95% confidence interval will be extracted from each study and combined. If the adjusted OR is not provided in the text or table, an OR will be calculated using the number of patients with and without the outcome in exposed and non-exposed groups. For studies that report multiple adjusted ORs, the OR adjusted for the greatest number of variables will be used in this meta-analysis.

Primary and secondary outcomes will be pooled with recorded data. Heterogeneity will be assessed with Q statistic and Higgens I^2 test. Q statistic ($P \geq 0.1$) and I^2-statistic (I^2 ＜30%) will be regarded as low heterogeneity [27]. Chi-square test will be used to combine data with 95% CI. A fixed model will be taken for data combination with low heterogeneity. Elsewise, a random effect model will be used. A two-tailed P value less than 0.05 will be considered as statistically significant.

Publication bias for the primary outcome will be assessed separately in randomized studies and observational studies using funnel plots and Egger's test.

Subgroup and Sensitivity Analyses

1) Baseline illness severity of studied patients
2) Antiplatelet drug
3) Timing of antiplatelet therapy
4) Combined anticoagulation therapy
5) Study centers
6) Original effect size

All statistical analyses will be performed in RStudio (Version 1.1.463).

Reporting of Results

Results will be reported according to the Preferred Reporting Items for Systematic Reviews and Meta-Analyses (PRISMA) (http://www.prisma-statement.org)[25].

Reference

1. Bikdeli B, Madhavan MV, Jimenez D, Chuich T, Dreyfus I, Driggin E, Nigoghossian C, Ageno W, Madjid M, Guo Y *et al*: **COVID-19 and Thrombotic or Thromboembolic Disease: Implications for Prevention, Antithrombotic Therapy, and Follow-Up: JACC State-of-the-Art Review**. *J Am Coll Cardiol* 2020, **75**(23):2950-2973.
2. Lazzaroni MG, Piantoni S, Masneri S, Garrafa E, Martini G, Tincani A, Andreoli L, Franceschini F: **Coagulation dysfunction in COVID-19: The interplay between inflammation, viral infection and the coagulation system**. *Blood Rev* 2021, **46**:100745.
3. van der Meijden PEJ, Heemskerk JWM: **Platelet biology and functions: new concepts and clinical perspectives**. *Nat Rev Cardiol* 2019, **16**(3):166-179.
4. Martinod K, Deppermann C: **Immunothrombosis and thromboinflammation in host defense and disease**. *Platelets* 2021, **32**(3):314-324.
5. Clark SR, Ma AC, Tavener SA, McDonald B, Goodarzi Z, Kelly MM, Patel KD, Chakrabarti S, McAvoy E, Sinclair GD *et al*: **Platelet TLR4 activates neutrophil extracellular traps to ensnare bacteria in septic blood**. *Nat Med* 2007, **13**(4):463-469.
6. Middleton EA, Weyrich AS, Zimmerman GA: **Platelets in Pulmonary Immune Responses and Inflammatory Lung Diseases**. *Physiol Rev* 2016, **96**(4):1211-1259.

7. McDonald B, Davis RP, Kim SJ, Tse M, Esmon CT, Kolaczkowska E, Jenne CN: **Platelets and neutrophil extracellular traps collaborate to promote intravascular coagulation during sepsis in mice**. *Blood* 2017, **129**(10):1357-1367.
8. Barrett TJ, Lee AH, Xia Y, Lin LH, Black M, Cotzia P, Hochman J, Berger JS: **Platelet and Vascular Biomarkers Associate With Thrombosis and Death in Coronavirus Disease**. *Circ Res* 2020, **127**(7):945-947.
9. Taus F, Salvagno G, Canè S, Fava C, Mazzaferri F, Carrara E, Petrova V, Barouni RM, Dima F, Dalbeni A *et al*: **Platelets Promote Thromboinflammation in SARS-CoV-2 Pneumonia**. *Arterioscler Thromb Vasc Biol* 2020, **40**(12):2975-2989.
10. Amison RT, O'Shaughnessy BG, Arnold S, Cleary SJ, Nandi M, Pitchford SC, Bragonzi A, Page CP: **Platelet Depletion Impairs Host Defense to Pulmonary Infection with Pseudomonas aeruginosa in Mice**. *Am J Respir Cell Mol Biol* 2018, **58**(3):331-340.
11. Hottz ED, Azevedo-Quintanilha IG, Palhinha L, Teixeira L, Barreto EA, Pão CRR, Righy C, Franco S, Souza TML, Kurtz P *et al*: **Platelet activation and platelet-monocyte aggregate formation trigger tissue factor expression in patients with severe COVID-19**. *Blood* 2020, **136**(11):1330-1341.
12. Middleton EA, He XY, Denorme F, Campbell RA, Ng D, Salvatore SP, Mostyka M, Baxter-Stoltzfus A, Borczuk AC, Loda M *et al*: **Neutrophil extracellular traps contribute to immunothrombosis in COVID-19 acute respiratory distress syndrome**. *Blood* 2020, **136**(10):1169-1179.
13. Denorme F, Ajanel A, Campbell RA: **Shining a light on platelet activation in COVID-19**. *J Thromb Haemost* 2022.
14. Bianconi V, Violi F, Fallarino F, Pignatelli P, Sahebkar A, Pirro M: **Is Acetylsalicylic Acid a Safe and Potentially Useful Choice for Adult Patients with COVID-19 ?** *Drugs* 2020, **80**(14):1383-1396.
15. Berger JS, Kornblith LZ, Gong MN, Reynolds HR, Cushman M, Cheng Y, McVerry BJ, Kim KS, Lopes RD, Atassi B *et al*: **Effect of P2Y12 Inhibitors on Survival Free of Organ Support Among Non–Critically Ill Hospitalized Patients With COVID-19**. *Jama* 2022, **327**(3).
16. Aydınyılmaz F, Aksakal E, Pamukcu HE, Aydemir S, Doğan R, Saraç İ, Aydın S, Kalkan K, Gülcü O, Tanboğa İ H: **Significance of MPV, RDW and PDW with the Severity and Mortality of COVID-19 and Effects of Acetylsalicylic Acid Use**. *Clin Appl Thromb Hemost* 2021, **27**:10760296211048808.
17. Gupta A, Madhavan MV, Poterucha TJ, DeFilippis EM, Hennessey JA, Redfors B, Eckhardt C, Bikdeli B, Platt J, Nalbandian A *et al*: **Association between antecedent statin use and decreased mortality in hospitalized patients with COVID-19**. *Nat Commun* 2021, **12**(1):1325.
18. Meizlish ML, Goshua G, Liu Y, Fine R, Amin K, Chang E, DeFilippo N, Keating C, Liu Y, Mankbadi M *et al*: **Intermediate-dose anticoagulation, aspirin, and in-hospital mortality in COVID-19: A propensity score-matched analysis**. *Am J Hematol* 2021, **96**(4):471-479.
19. Osborne TF, Veigulis ZP, Arreola DM, Mahajan SM, Roosli E, Curtin CM: **Association of mortality and aspirin prescription for COVID-19 patients at the Veterans Health Administration**. *PLoS ONE* 2021, **16**(2 February).
20. Santoro F, Nuñez-Gil IJ, Vitale E, Viana-Llamas MC, Reche-Martinez B, Romero-Pareja R, Feltez Guzman G, Fernandez Rozas I, Uribarri A, Becerra-Muñoz VM *et al*: **Antiplatelet**

therapy and outcome in COVID-19: the Health Outcome Predictive Evaluation Registry. *Heart* 2021.

21. Zhao X, Gao C, Dai F, Treggiari MM, Deshpande R, Meng L: **Treatments Associated with Lower Mortality among Critically Ill COVID-19 Patients**. *Anesthesiology* 2021.
22. Abani O, Abbas A, Abbas F, Abbas M, Abbasi S, Abbass H, Abbott A, Abdallah N, Abdelaziz A, Abdelfattah M *et al*: **Aspirin in patients admitted to hospital with COVID-19 (RECOVERY): a randomised, controlled, open-label, platform trial**. *The Lancet* 2022, **399**(10320):143-151.
23. Investigators R-CWCftR-C, Bradbury CA, Lawler PR, Stanworth SJ, McVerry BJ, McQuilten Z, Higgins AM, Mouncey PR, Al-Beidh F, Rowan KM *et al*: **Effect of Antiplatelet Therapy on Survival and Organ Support-Free Days in Critically Ill Patients With COVID-19: A Randomized Clinical Trial**. *JAMA* 2022.
24. Higgins JP, Altman DG, Gotzsche PC et al. **The Cochrane Collaboration's tool for assessing risk of bias in randomised trials**. *BMJ.* 2011; 343:d5928..
25. Moher D, Liberati A, Tetzlaff J, Altman DG, Group P: **Preferred reporting items for systematic reviews and meta-analyses: the PRISMA statement**. *BMJ* 2009, **339**:b2535.
26. Guyatt G, Oxman AD, Akl EA et al. **GRADE guidelines: 1. Introduction-GRADE evidence profiles and summary of findings tables**. *J Clin Epidemiol*. 2011; 64(4):383-94..
27. Higgins JP, Thompson SG, Deeks JJ, Altman DG: **Measuring inconsistency in meta-analyses**. *Bmj* 2003, **327**(7414):557-560.
