# Supplementary material for: Antiplatelet therapy for patients with COVID-19: Systematic review and meta-analysis of observational studies and randomized controlled trials
Source: Front Med (Lausanne). 2022 Sep 7;9:965790. doi: 10.3389/fmed.2022.965790 (PMC9490267; doi:10.3389/fmed.2022.965790)
Supplement: Supplementary file 2 [file Data_Sheet_2.pdf]

*Supplementary file 2*

**This word file inculdes:**

**Table S1** Quality assessment of the included observational studies by Newcastle-Ottawa scale

**Figure S1** Sensitivity analysis of pooled analysis

**Figure S2** Subgroup analysis of the included studies based on original effect size

**Figure S3** Subgroup analysis of the included studies based on study center

**Figure S4** Subgroup analysis of the included studies based on illness severity

**Figure S5** Subgroup analysis of the included studies based on antiplatelet agents

**Figure S6** Subgroup analysis of the included studies based on treatment timing

**Figure S7** Subgroup analysis of the included studies based on anticoagulants use

**Figure S8** Funnel plot for publication bias tests

## *Supplementary Table and Figures*

**Table S1 |** Quality assessment of the included observational studies by the Newcastle–Ottawa scale

| Cohort Studies | | | | | | | | | |
|---|---|---|---|---|---|---|---|---|---|
| **Study** | **Selection** | | | | **Comparability** | **Outcome** | | | **Total score** |
| | Exposed Cohort | Nonexposed Cohort | Ascertainment of exposure | Outcome of interest | | Assessment of outcome | Length of follow-up | Adequacy of follow-up | |
| Aydınyılmaz (41) | 1 | 1 | 0 | 1 | 1 | 1 | 1 | 1 | 7 |
| Chow (42) | 1 | 1 | 1 | 1 | 1 | 1 | 1 | 1 | 8 |
| Corrochano (43) | 1 | 1 | 1 | 1 | 1 | 1 | 1 | 1 | 8 |
| Fröhlich (44) | 1 | 1 | 1 | 1 | 1 | 1 | 1 | 1 | 8 |
| Gupta (45) | 1 | 1 | 0 | 1 | 1 | 1 | 1 | 1 | 7 |
| Aghajani (46) | 1 | 1 | 1 | 1 | 1 | 1 | 0 | 1 | 7 |
| Ho (47) | 1 | 1 | 1 | 1 | 1 | 1 | 1 | 1 | 8 |
| Izzi-Engbeaya (48) | 1 | 0 | 0 | 1 | 1 | 1 | 1 | 1 | 6 |
| Liu (49) | 1 | 1 | 1 | 1 | 1 | 1 | 1 | 1 | 8 |
| Matli (50) | 1 | 1 | 1 | 1 | 1 | 1 | 1 | 1 | 8 |
| Meizlish (51) | 1 | 1 | 1 | 1 | 1 | 1 | 1 | 1 | 8 |
| Merzon (52) | 1 | 1 | 1 | 1 | 1 | 1 | 0 | 1 | 7 |
| Mura (53) | 1 | 1 | 1 | 1 | 1 | 1 | 0 | 0 | 6 |
| Osborne (54) | 1 | 1 | 1 | 1 | 1 | 1 | 1 | 1 | 8 |
| Pan (55) | 1 | 1 | 1 | 1 | 1 | 1 | 1 | 1 | 8 |
| Russo (56) | 1 | 1 | 1 | 1 | 1 | 1 | 1 | 1 | 8 |
| Sahai (57) | 1 | 1 | 1 | 1 | 1 | 1 | 0 | 1 | 7 |
| Santoro (58) | 1 | 1 | 1 | 1 | 1 | 1 | 1 | 1 | 8 |
| Sisinni (59) | 1 | 1 | 1 | 1 | 1 | 1 | 1 | 1 | 8 |
| Soldevila (60) | 1 | 1 | 1 | 1 | 1 | 1 | 1 | 1 | 8 |
| Terlecki (61) | 1 | 1 | 1 | 1 | 1 | 1 | 1 | 1 | 8 |
| Tremblay (62) | 1 | 1 | 1 | 1 | 1 | 1 | 1 | 1 | 8 |
| Zhao (63) | 1 | 1 | 1 | 1 | 1 | 1 | 1 | 1 | 8 |

**References**


41. Aydınyılmaz F, Aksakal E, Pamukcu HE, Aydemir S, Doğan R, Saraç İ, et al. Significance of MPV, RDW and PDW with the Severity and Mortality of COVID-19 and Effects of Acetylsalicylic Acid Use. *Clin Appl Thromb Hemost*. (2021) 27: 10760296211048808. doi:10.1177/10760296211048808
42. Chow JH, Yin Y, Yamane DP, Davison D, Keneally RJ, Hawkins K, et al. Association of prehospital antiplatelet therapy with survival in patients hospitalized with COVID-19: A propensity score-matched analysis. *J Thromb Haemost*. (2021) 19: 2814-24. doi:10.1111/jth.15517
43. Corrochano M, Acosta-Isaac R, Mojal S, Miqueleiz S, Rodriguez D, Quijada-Manuitt M, et al. Impact of pre-admission antithrombotic therapy on disease severity and mortality in patients

hospitalized for COVID-19. *J Thromb Thrombolysis*. (2021): 1-7. doi:10.1007/s11239-021-02507-2
44. Fröhlich GM, Jeschke E, Eichler U, Thiele H, Alhariri L, Reinthaler M, et al. Impact of oral anticoagulation on clinical outcomes of COVID-19: a nationwide cohort study of hospitalized patients in Germany. *Clin Res Cardiol*. (2021) 110: 1041-50. doi:10.1007/s00392-020-01783-x
45. Gupta A, Madhavan MV, Poterucha TJ, Defilippis EM, Hennessey JA, Redfors B, et al. Association between antecedent statin use and decreased mortality in hospitalized patients with COVID-19. *Nat Commun*. (2021) 12: 1325. doi:10.1038/s41467-021-21553-1
46. Haji Aghajani M, Moradi O, Amini H, Azhdari Tehrani H, Pourheidar E, Rabiei MM, et al. Decreased in-hospital mortality associated with aspirin administration in hospitalized patients due to severe COVID-19. *J Med Virol*. (2021) 93: 5390-5. doi:10.1002/jmv.27053
47. Ho G, Dusendang JR, Schmittdiel J, Kavecansky J, Tavakoli J, Pai A. Association of chronic anticoagulant and antiplatelet use on disease severity in SARS-COV-2 infected patients. *J Thromb Thrombolysis*. (2021) 52: 476-81. doi:10.1007/s11239-021-02383-w
48. Izzi-Engbeaya C, Distaso W, Amin A, Yang W, Idowu O, Kenkre JS, et al. Adverse outcomes in COVID-19 and diabetes: a retrospective cohort study from three London teaching hospitals. *BMJ Open Diabetes Res Care*. (2021) 9. doi:10.1136/bmjdrc-2020-001858
49. Liu Q, Huang N, Li A, Zhou Y, Liang L, Song X, et al. Effect of low-dose aspirin on mortality and viral duration of the hospitalized adults with COVID-19. *Medicine (Baltimore)*. (2021) 100: e24544. doi:10.1097/md.0000000000024544
50. Matli K, Chamoun N, Fares A, Zibara V, Al-Osta S, Nasrallah R, et al. Combined anticoagulant and antiplatelet therapy is associated with an improved outcome in hospitalised patients with COVID-19: a propensity matched cohort study. *Open Heart*. (2021) 8. doi:10.1136/openhrt-2021-001785
51. Meizlish ML, Goshua G, Liu Y, Fine R, Amin K, Chang E, et al. Intermediate-dose anticoagulation, aspirin, and in-hospital mortality in COVID-19: A propensity score-matched analysis. *Am J Hematol*. (2021) 96: 471-9. doi:10.1002/ajh.26102
52. Merzon E, Green I, Vinker S, Golan-Cohen A, Gorohovski A, Avramovich E, et al. The use of aspirin for primary prevention of cardiovascular disease is associated with a lower likelihood of COVID-19 infection. *Febs j*. (2021) 288: 5179-89. doi:10.1111/febs.15784
53. Mura C, Preissner S, Nahles S, Heiland M, Bourne PE, Preissner R. Real-world evidence for improved outcomes with histamine antagonists and aspirin in 22,560 COVID-19 patients. *Signal Transduct Target Ther*. (2021) 6: 267. doi:10.1038/s41392-021-00689-y
54. Osborne TF, Veigulis ZP, Arreola DM, Mahajan SM, Roosli E, Curtin CM. Association of mortality and aspirin prescription for COVID-19 patients at the Veterans Health Administration. *PLoS ONE*. (2021) 16. doi:10.1371/journal.pone.0246825
55. Pan D, Ip A, Zhan S, Wasserman I, Snyder DJ, Agathis AZ, et al. Pre-hospital antiplatelet medication use on COVID-19 disease severity. *Heart Lung*. (2021) 50: 618-21. doi:10.1016/j.hrtlng.2021.04.010
56. Russo V, Di Maio M, Attena E, Silverio A, Scudiero F, Celentani D, et al. Clinical impact of pre-admission antithrombotic therapy in hospitalized patients with COVID-19: A multicenter observational study. *Pharmacol Res*. (2020) 159: 104965. doi:10.1016/j.phrs.2020.104965
57. Sahai A, Bhandari R, Godwin M, Mcintyre T, Chung MK, Iskandar JP, et al. Effect of aspirin on short-term outcomes in hospitalized patients with COVID-19. *Vasc Med*. (2021):

1358863x211012754. doi:10.1177/1358863x211012754
58. Santoro F, Nuñez-Gil IJ, Vitale E, Viana-Llamas MC, Reche-Martinez B, Romero-Pareja R, et al. Antiplatelet therapy and outcome in COVID-19: the Health Outcome Predictive Evaluation Registry. *Heart*. (2021). doi:10.1136/heartjnl-2021-319552
59. Sisinni A, Rossi L, Battista A, Poletti E, Battista F, Battista RA, et al. Pre-admission acetylsalicylic acid therapy and impact on in-hospital outcome in COVID-19 patients: The ASA-CARE study. *Int J Cardiol*. (2021) 344: 240-5. doi:10.1016/j.ijcard.2021.09.058
60. Soldevila L, Valerio-Sallent L, Roure S, Pérez-Quílez O, Mas M, Miralles R, et al. Drug exposure may have a substantial influence on COVID-19 prognosis among residents of long-term care facilities: an exploratory analysis. *Int J Infect Dis*. (2021) 109: 192-4. doi:10.1016/j.ijid.2021.07.007
61. Terlecki M, Wojciechowska W, Klocek M, Olszanecka A, Stolarz-Skrzypek K, Grodzicki T, et al. Association between cardiovascular disease, cardiovascular drug therapy, and in-hospital outcomes in patients with COVID-19: Data from a large single-center registry in Poland. *Kardiologia Polska*. (2021) 79: 773-80. doi:10.33963/KP.15990
62. Tremblay D, Van Gerwen M, Alsen M, Thibaud S, Kessler A, Venugopal S, et al. Impact of anticoagulation prior to COVID-19 infection: a propensity score-matched cohort study. *Blood*. (2020) 136: 144-7. doi:10.1182/blood.2020006941
63. Zhao X, Gao C, Dai F, Treggiari MM, Deshpande R, Meng L. Treatments Associated with Lower Mortality among Critically Ill COVID-19 Patients. *Anesthesiology*. (2021). doi:10.1097/aln.0000000000003999

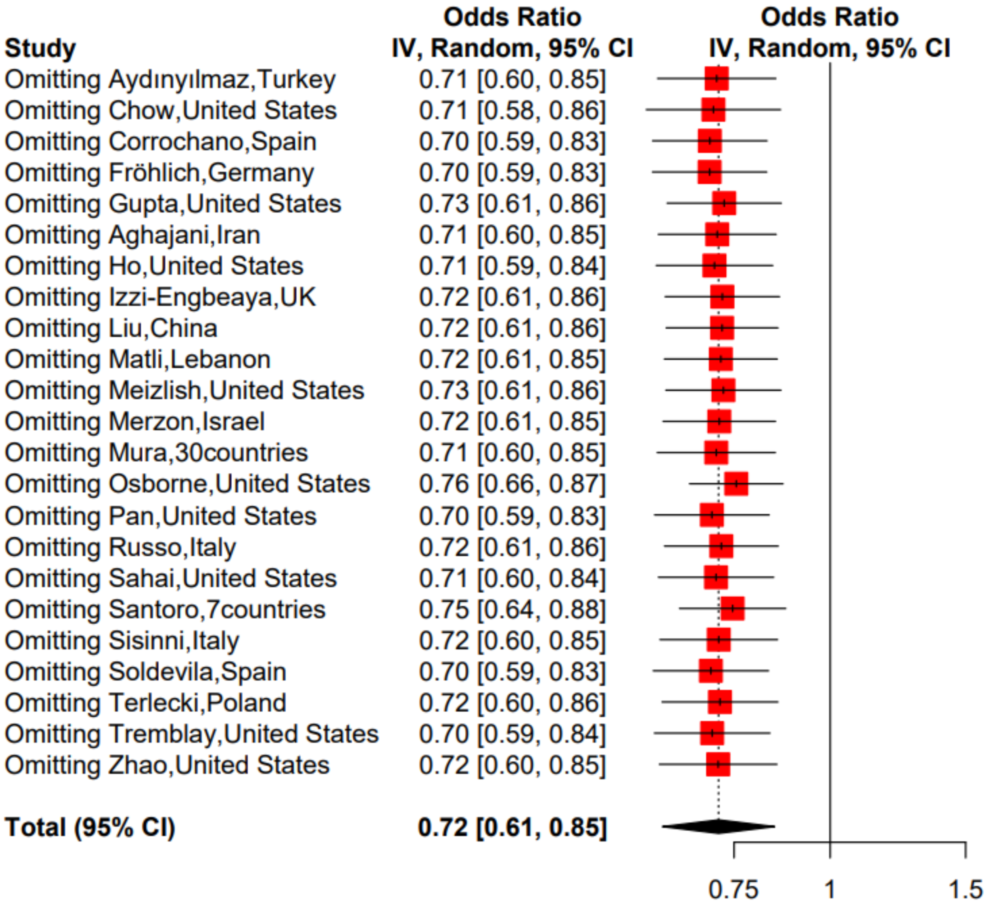


**Figure S1 |** Sensitivity analysis of the pooled analysis

| Study or Subgroup | TE | SE | Weight | Odds Ratio IV, Random, 95% CI |
|---|---|---|---|---|
| **Effect.size = HR** | | | | |
| Aydınyılmaz,Turkey | -0.26 | 0.1304 | 5.5% | 0.77 [0.60, 1.00] |
| Chow,United States | -0.21 | 0.0345 | 6.3% | 0.81 [0.76, 0.87] |
| Aghajani,Iran | -0.28 | 0.1398 | 5.4% | 0.75 [0.57, 0.99] |
| Matli,Lebanon | -0.89 | 0.7051 | 1.2% | 0.41 [0.10, 1.63] |
| Meizlish,United States | -0.65 | 0.2251 | 4.4% | 0.52 [0.34, 0.81] |
| Santoro,7countries | -0.94 | 0.1034 | 5.8% | 0.39 [0.32, 0.48] |
| Sisinni,Italy | -0.36 | 0.1442 | 5.4% | 0.70 [0.53, 0.92] |
| Tremblay,United States | 0.03 | 0.1803 | 4.9% | 1.03 [0.72, 1.47] |
| Zhao,United States | -0.33 | 0.1468 | 5.3% | 0.72 [0.54, 0.96] |
| **Total (95% CI)** | | | **44.2%** | **0.68 [0.55, 0.84]** |
| Heterogeneity: $Tau^2 = 0.08$; $Chi^2 = 51.74$, df = 8 ($P < 0.01$); $I^2 = 85\%$ | | | | |
| Test for overall effect: $Z = -3.57$ ($P < 0.01$) | | | | |
| **Effect.size = OR** | | | | |
| Corrochano,Spain | 0.17 | 0.1738 | 5.0% | 1.18 [0.84, 1.66] |
| Fröhlich,Germany | 0.10 | 0.0854 | 6.0% | 1.10 [0.93, 1.30] |
| Gupta,United States | -0.77 | 0.2860 | 3.7% | 0.46 [0.26, 0.81] |
| Ho,United States | -0.12 | 0.1687 | 5.1% | 0.89 [0.64, 1.24] |
| Izzi-Engbeaya,UK | -0.66 | 0.3190 | 3.4% | 0.52 [0.28, 0.96] |
| Liu,China | -2.24 | 1.1128 | 0.5% | 0.11 [0.01, 0.94] |
| Merzon,Israel | -1.02 | 1.4888 | 0.3% | 0.36 [0.02, 6.66] |
| Mura,30countries | -0.24 | 0.1311 | 5.5% | 0.79 [0.61, 1.02] |
| Osborne,United States | -0.97 | 0.0791 | 6.0% | 0.38 [0.33, 0.44] |
| Pan,United States | 0.12 | 0.2438 | 4.2% | 1.13 [0.70, 1.82] |
| Russo,Italy | -0.67 | 0.4338 | 2.4% | 0.51 [0.22, 1.19] |
| Sahai,United States | -0.16 | 0.2594 | 4.0% | 0.85 [0.51, 1.41] |
| Soldevila,Spain | 0.09 | 0.1604 | 5.2% | 1.09 [0.80, 1.49] |
| Terlecki,Poland | -0.43 | 0.2173 | 4.5% | 0.65 [0.42, 0.99] |
| **Total (95% CI)** | | | **55.8%** | **0.73 [0.54, 0.98]** |
| Heterogeneity: $Tau^2 = 0.24$; $Chi^2 = 118.06$, df = 13 ($P < 0.01$); $I^2 = 89\%$ | | | | |
| Test for overall effect: $Z = -2.07$ ($P = 0.04$) | | | | |
| **Total (95% CI)** | | | **100.0%** | **0.72 [0.61, 0.85]** |

Heterogeneity: $Tau^2 = 0.12$; $Chi^2 = 170.66$, df = 22 ($P < 0.01$); $I^2 = 87\%$
Residual heterogeneity: $Tau^2$ = NA; $Chi^2 = 169.80$, df = 21 ($P < 0.01$); $I^2 = 88\%$
Test for overall effect: $Z = -3.90$ ($P < 0.01$)
Test for subgroup differences: $Chi^2 = 0.15$, df = 1 ($P = 0.70$)

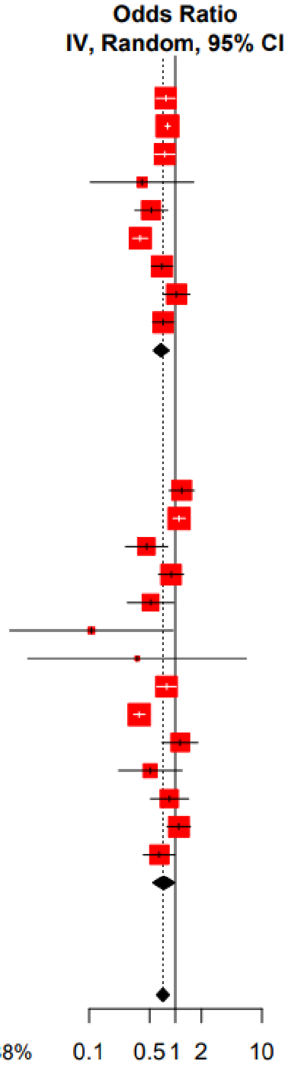


**Figure S2 |** Subgroup analysis of the included studies based on the original effect size

| Study or Subgroup | TE | SE | Weight | Odds Ratio IV, Random, 95% CI |
|---|---|---|---|---|
| **Center = M** | | | | |
| Chow,United States | -0.21 | 0.0345 | 6.3% | 0.81 [0.76, 0.87] |
| Fröhlich,Germany | 0.10 | 0.0854 | 6.0% | 1.10 [0.93, 1.30] |
| Ho,United States | -0.12 | 0.1687 | 5.1% | 0.89 [0.64, 1.24] |
| Izzi-Engbeaya,UK | -0.66 | 0.3190 | 3.4% | 0.52 [0.28, 0.96] |
| Meizlish,United States | -0.65 | 0.2251 | 4.4% | 0.52 [0.34, 0.81] |
| Merzon,Israel | -1.02 | 1.4888 | 0.3% | 0.36 [0.02, 6.66] |
| Mura,30countries | -0.24 | 0.1311 | 5.5% | 0.79 [0.61, 1.02] |
| Osborne,United States | -0.97 | 0.0791 | 6.0% | 0.38 [0.33, 0.44] |
| Russo,Italy | -0.67 | 0.4338 | 2.4% | 0.51 [0.22, 1.19] |
| Sahai,United States | -0.16 | 0.2594 | 4.0% | 0.85 [0.51, 1.41] |
| Santoro,7countries | -0.94 | 0.1034 | 5.8% | 0.39 [0.32, 0.48] |
| Sisinni,Italy | -0.36 | 0.1442 | 5.4% | 0.70 [0.53, 0.92] |
| Soldevila,Spain | 0.09 | 0.1604 | 5.2% | 1.09 [0.80, 1.49] |
| Tremblay,United States | 0.03 | 0.1803 | 4.9% | 1.03 [0.72, 1.47] |
| Zhao,United States | -0.33 | 0.1468 | 5.3% | 0.72 [0.54, 0.96] |
| **Total (95% CI)** | | | **69.9%** | **0.70 [0.57, 0.87]** |
| Heterogeneity: $Tau^2 = 0.13$; $Chi^2 = 153.32$, df = 14 ($P < 0.01$); $I^2 = 91\%$ | | | | |
| Test for overall effect: $Z = -3.25$ ($P < 0.01$) | | | | |
| **Center = S** | | | | |
| Aydınyılmaz,Turkey | -0.26 | 0.1304 | 5.5% | 0.77 [0.60, 1.00] |
| Corrochano,Spain | 0.17 | 0.1738 | 5.0% | 1.18 [0.84, 1.66] |
| Gupta,United States | -0.77 | 0.2860 | 3.7% | 0.46 [0.26, 0.81] |
| Aghajani,Iran | -0.28 | 0.1398 | 5.4% | 0.75 [0.57, 0.99] |
| Liu,China | -2.24 | 1.1128 | 0.5% | 0.11 [0.01, 0.94] |
| Matli,Lebanon | -0.89 | 0.7051 | 1.2% | 0.41 [0.10, 1.63] |
| Pan,United States | 0.12 | 0.2438 | 4.2% | 1.13 [0.70, 1.82] |
| Terlecki,Poland | -0.43 | 0.2173 | 4.5% | 0.65 [0.42, 0.99] |
| **Total (95% CI)** | | | **30.1%** | **0.77 [0.60, 0.98]** |
| Heterogeneity: $Tau^2 = 0.06$; $Chi^2 = 16.09$, df = 7 ($P = 0.02$); $I^2 = 56\%$ | | | | |
| Test for overall effect: $Z = -2.12$ ($P = 0.03$) | | | | |
| **Total (95% CI)** | | | **100.0%** | **0.72 [0.61, 0.85]** |

Heterogeneity: $Tau^2 = 0.12$; $Chi^2 = 170.66$, df = 22 ($P < 0.01$); $I^2 = 87\%$
Residual heterogeneity: $Tau^2 = NA$; $Chi^2 = 169.41$, df = 21 ($P < 0.01$); $I^2 = 88\%$
Test for overall effect: $Z = -3.90$ ($P < 0.01$)
Test for subgroup differences: $Chi^2 = 0.30$, df = 1 ($P = 0.58$)

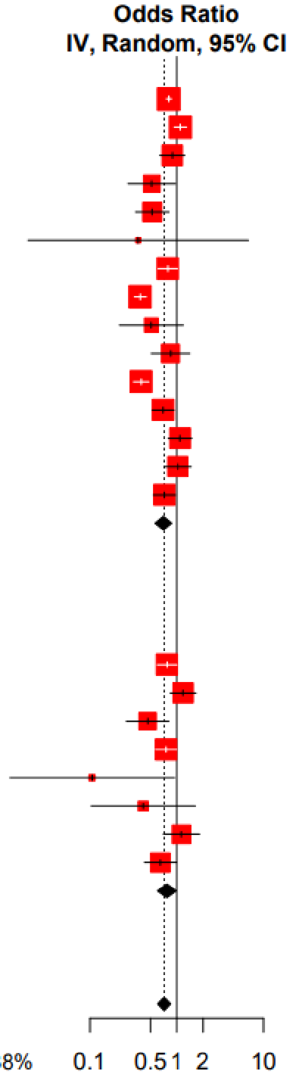


**Figure S3 |** Subgroup analysis of the included studies based on the study center

| Study or Subgroup | TE | SE | Weight | Odds Ratio IV, Random, 95% CI |
|---|---|---|---|---|
| **Illness.severity = All** | | | | |
| Chow,United States | -0.21 | 0.0345 | 6.3% | 0.81 [0.76, 0.87] |
| Corrochano,Spain | 0.17 | 0.1738 | 5.0% | 1.18 [0.84, 1.66] |
| Fröhlich,Germany | 0.10 | 0.0854 | 6.0% | 1.10 [0.93, 1.30] |
| Gupta,United States | -0.77 | 0.2860 | 3.7% | 0.46 [0.26, 0.81] |
| Ho,United States | -0.12 | 0.1687 | 5.1% | 0.89 [0.64, 1.24] |
| Izzi-Engbeaya,UK | -0.66 | 0.3190 | 3.4% | 0.52 [0.28, 0.96] |
| Liu,China | -2.24 | 1.1128 | 0.5% | 0.11 [0.01, 0.94] |
| Matli,Lebanon | -0.89 | 0.7051 | 1.2% | 0.41 [0.10, 1.63] |
| Meizlish,United States | -0.65 | 0.2251 | 4.4% | 0.52 [0.34, 0.81] |
| Merzon,Israel | -1.02 | 1.4888 | 0.3% | 0.36 [0.02, 6.66] |
| Osborne,United States | -0.97 | 0.0791 | 6.0% | 0.38 [0.33, 0.44] |
| Pan,United States | 0.12 | 0.2438 | 4.2% | 1.13 [0.70, 1.82] |
| Russo,Italy | -0.67 | 0.4338 | 2.4% | 0.51 [0.22, 1.19] |
| Sahai,United States | -0.16 | 0.2594 | 4.0% | 0.85 [0.51, 1.41] |
| Santoro,7countries | -0.94 | 0.1034 | 5.8% | 0.39 [0.32, 0.48] |
| Sisinni,Italy | -0.36 | 0.1442 | 5.4% | 0.70 [0.53, 0.92] |
| Soldevila,Spain | 0.09 | 0.1604 | 5.2% | 1.09 [0.80, 1.49] |
| Terlecki,Poland | -0.43 | 0.2173 | 4.5% | 0.65 [0.42, 0.99] |
| Tremblay,United States | 0.03 | 0.1803 | 4.9% | 1.03 [0.72, 1.47] |
| **Total (95% CI)** | | | **78.2%** | **0.70 [0.57, 0.86]** |
| Heterogeneity: $Tau^2 = 0.15$; $Chi^2 = 170.22$, df = 18 ($P < 0.01$); $I^2 = 89\%$ | | | | |
| Test for overall effect: $Z = -3.32$ ($P < 0.01$) | | | | |
| **Illness.severity = Severe** | | | | |
| Aydınyılmaz,Turkey | -0.26 | 0.1304 | 5.5% | 0.77 [0.60, 1.00] |
| Aghajani,Iran | -0.28 | 0.1398 | 5.4% | 0.75 [0.57, 0.99] |
| Mura,30countries | -0.24 | 0.1311 | 5.5% | 0.79 [0.61, 1.02] |
| Zhao,United States | -0.33 | 0.1468 | 5.3% | 0.72 [0.54, 0.96] |
| **Total (95% CI)** | | | **21.8%** | **0.76 [0.67, 0.87]** |
| Heterogeneity: $Tau^2 = 0$; $Chi^2 = 0.24$, df = 3 ($P = 0.97$); $I^2 = 0\%$ | | | | |
| Test for overall effect: $Z = -4.00$ ($P < 0.01$) | | | | |
| **Total (95% CI)** | | | **100.0%** | **0.72 [0.61, 0.85]** |

Heterogeneity: $Tau^2 = 0.12$; $Chi^2 = 170.66$, df = 22 ($P < 0.01$); $I^2 = 87\%$
Residual heterogeneity: $Tau^2$ = NA; $Chi^2 = 170.47$, df = 21 ($P < 0.01$); $I^2 = 88\%$
Test for overall effect: $Z = -3.90$ ($P < 0.01$)
Test for subgroup differences: $Chi^2 = 0.42$, df = 1 ($P = 0.51$)

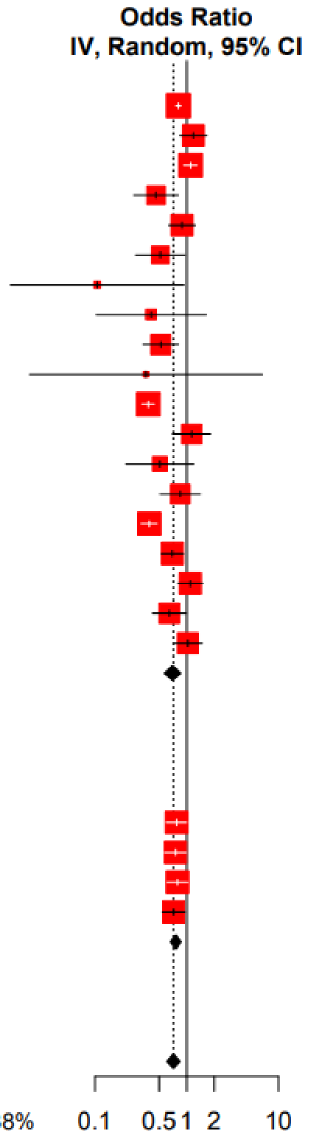


**Figure S4 |** Subgroup analysis of the included studies based on illness severity

| Study or Subgroup | TE | SE | Weight | Odds Ratio IV, Random, 95% CI |
|---|---|---|---|---|
| **Drugs = aspirin** | | | | |
| Aydınyılmaz,Turkey | -0.26 | 0.1304 | 5.5% | 0.77 [0.60, 1.00] |
| Aghajani,Iran | -0.28 | 0.1398 | 5.4% | 0.75 [0.57, 0.99] |
| Liu,China | -2.24 | 1.1128 | 0.5% | 0.11 [0.01, 0.94] |
| Meizlish,United States | -0.65 | 0.2251 | 4.4% | 0.52 [0.34, 0.81] |
| Merzon,Israel | -1.02 | 1.4888 | 0.3% | 0.36 [0.02, 6.66] |
| Mura,30countries | -0.24 | 0.1311 | 5.5% | 0.79 [0.61, 1.02] |
| Osborne,United States | -0.97 | 0.0791 | 6.0% | 0.38 [0.33, 0.44] |
| Sahai,United States | -0.16 | 0.2594 | 4.0% | 0.85 [0.51, 1.41] |
| Zhao,United States | -0.33 | 0.1468 | 5.3% | 0.72 [0.54, 0.96] |
| **Total (95% CI)** | | | **37.0%** | **0.63 [0.47, 0.85]** |
| Heterogeneity: $Tau^2 = 0.13$; $Chi^2 = 49.32$, df = 8 ($P < 0.01$); $I^2 = 84\%$ | | | | |
| Test for overall effect: Z = -3.10 ($P < 0.01$) | | | | |
| **Drugs = multiple** | | | | |
| Chow,United States | -0.21 | 0.0345 | 6.3% | 0.81 [0.76, 0.87] |
| Corrochano,Spain | 0.17 | 0.1738 | 5.0% | 1.18 [0.84, 1.66] |
| Fröhlich,Germany | 0.10 | 0.0854 | 6.0% | 1.10 [0.93, 1.30] |
| Ho,United States | -0.12 | 0.1687 | 5.1% | 0.89 [0.64, 1.24] |
| Matli,Lebanon | -0.89 | 0.7051 | 1.2% | 0.41 [0.10, 1.63] |
| Pan,United States | 0.12 | 0.2438 | 4.2% | 1.13 [0.70, 1.82] |
| Russo,Italy | -0.67 | 0.4338 | 2.4% | 0.51 [0.22, 1.19] |
| Santoro,7countries | -0.94 | 0.1034 | 5.8% | 0.39 [0.32, 0.48] |
| Sisinni,Italy | -0.36 | 0.1442 | 5.4% | 0.70 [0.53, 0.92] |
| Soldevila,Spain | 0.09 | 0.1604 | 5.2% | 1.09 [0.80, 1.49] |
| Terlecki,Poland | -0.43 | 0.2173 | 4.5% | 0.65 [0.42, 0.99] |
| **Total (95% CI)** | | | **50.9%** | **0.80 [0.64, 0.99]** |
| Heterogeneity: $Tau^2 = 0.10$; $Chi^2 = 77.2$, df = 10 ($P < 0.01$); $I^2 = 87\%$ | | | | |
| Test for overall effect: Z = -2.01 (P = 0.04) | | | | |
| **Drugs = NR** | | | | |
| Izzi-Engbeaya,UK | -0.66 | 0.3190 | 3.4% | 0.52 [0.28, 0.96] |
| Tremblay,United States | 0.03 | 0.1803 | 4.9% | 1.03 [0.72, 1.47] |
| **Total (95% CI)** | | | **8.3%** | **0.77 [0.39, 1.50]** |
| Heterogeneity: $Tau^2 = 0.17$; $Chi^2 = 3.55$, df = 1 (P = 0.06); $I^2 = 72\%$ | | | | |
| Test for overall effect: Z = -0.78 (P = 0.44) | | | | |
| **Drugs = P2Y12 inhibitor** | | | | |
| Gupta,United States | -0.77 | 0.2860 | 3.7% | 0.46 [0.26, 0.81] |
| **Total (95% CI)** | | | **3.7%** | **0.46 [0.26, 0.81]** |
| Heterogeneity: not applicable | | | | |
| Test for overall effect: Z = -2.70 ($P < 0.01$) | | | | |
| **Total (95% CI)** | | | **100.0%** | **0.72 [0.61, 0.85]** |
| Heterogeneity: $Tau^2 = 0.12$; $Chi^2 = 170.66$, df = 22 ($P < 0.01$); $I^2 = 87\%$ | | | | |
| Residual heterogeneity: $Tau^2$ = NA; $Chi^2 = 130.07$, df = 19 ($P < 0.01$); $I^2 = 85\%$ | | | | |
| Test for overall effect: Z = -3.90 ($P < 0.01$) | | | | |
| Test for subgroup differences: $Chi^2 = 4.00$, df = 3 (P = 0.26) | | | | |

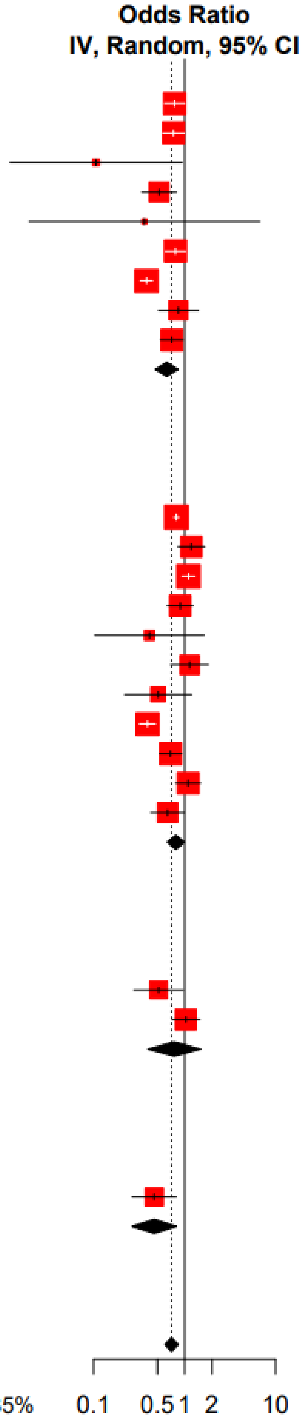


**Figure S5 |** Subgroup analysis of the included studies based on antiplatelet agents

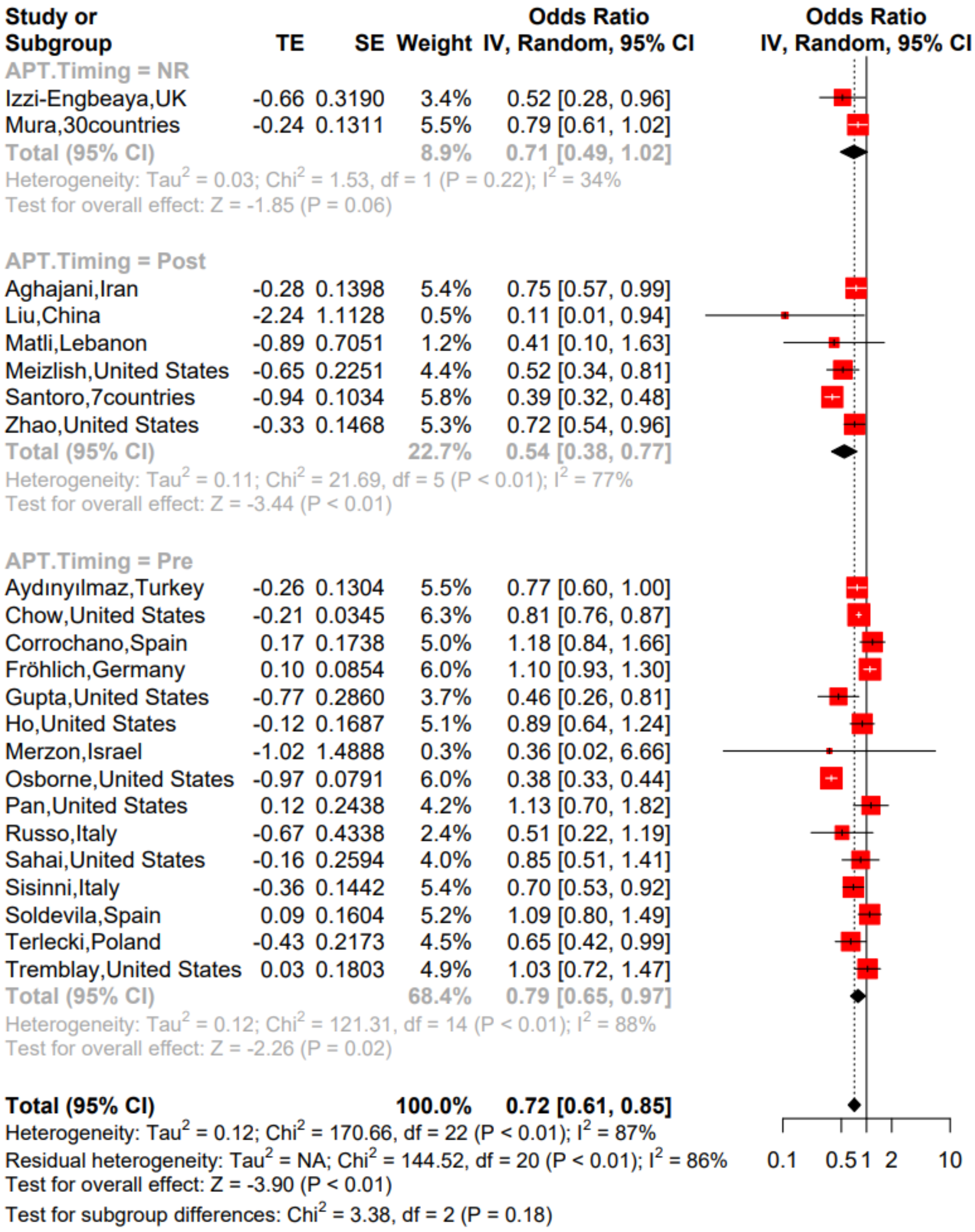


**Figure S6 |** Subgroup analysis of the included studies based on treatment timing

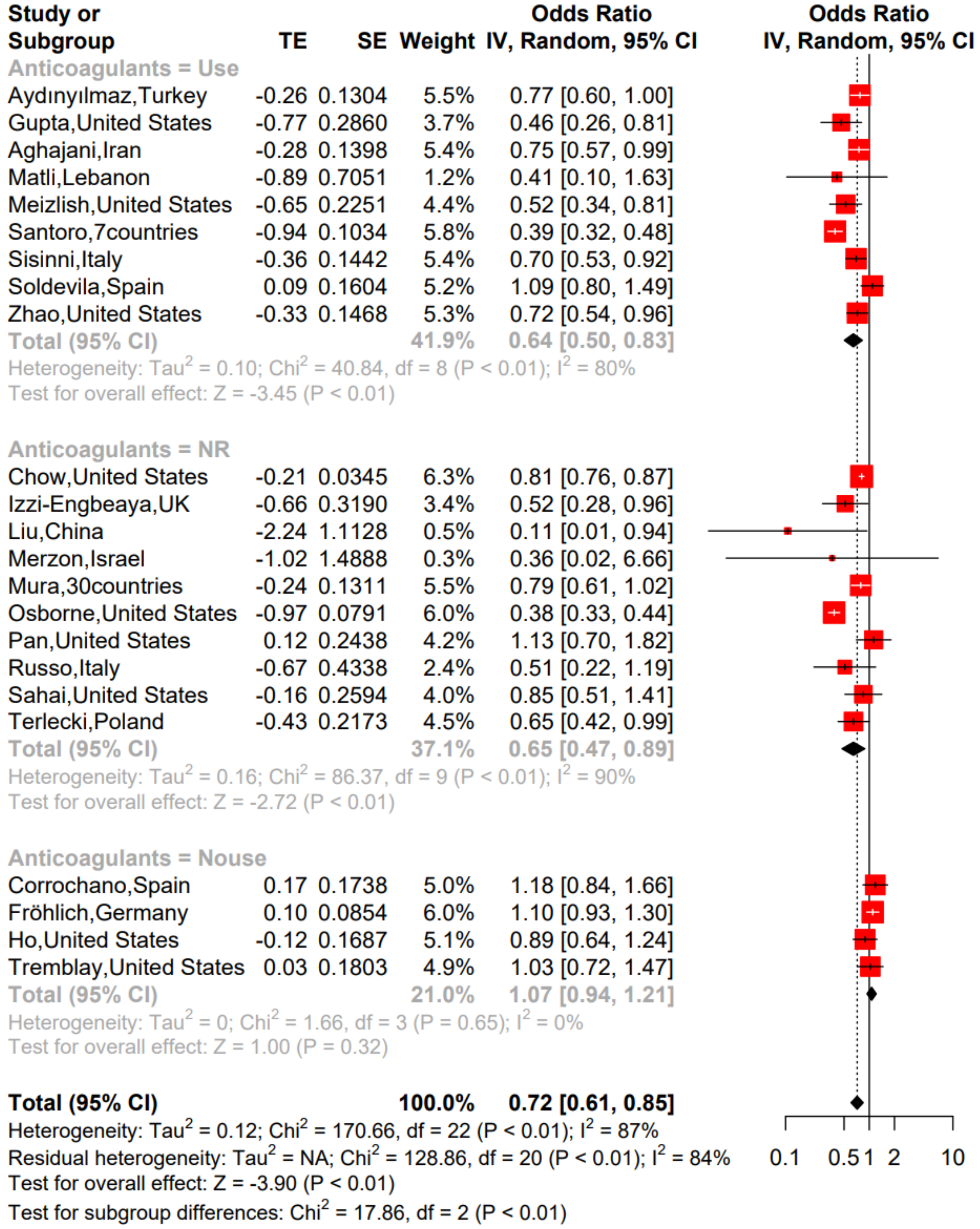


**Figure S7 |** Subgroup analysis of the included studies based on anticoagulant use

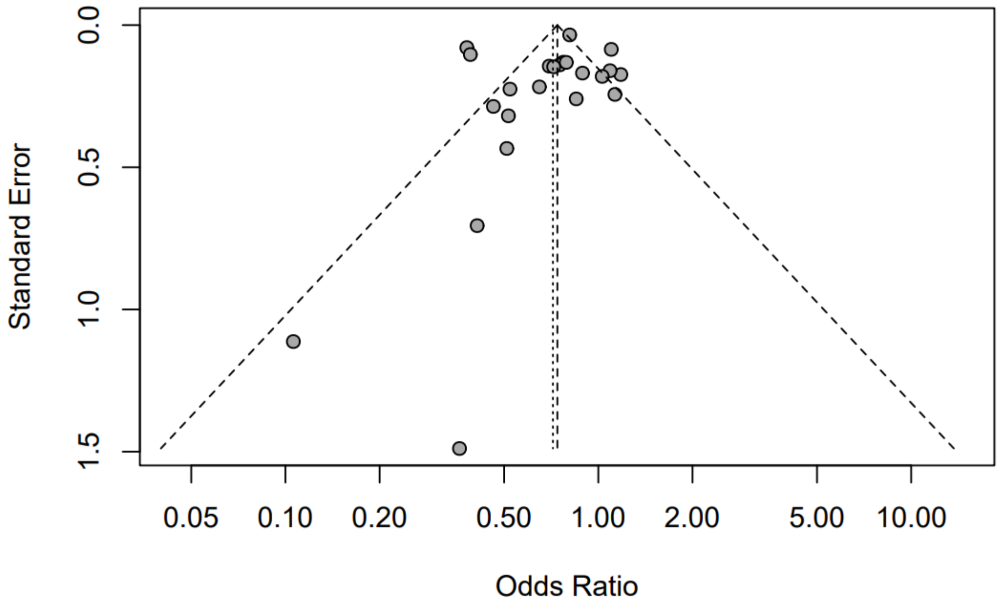


**Figure S8 |** Funnel plot for the publication bias tests
